# DoseRAD2026: A benchmark dataset for AI‐accelerated photon and proton dose calculation in radiotherapy

**DOI:** 10.1002/mp.70714

**Published:** 2026-10-26

**Authors:** Fan Xiao, Nikolaos Delopoulos, Niklas Wahl, Lennart Volz, Lina Bucher, Matteo Maspero, Miguel Palacios, Muheng Li, Samir Schulz, Viktor Rogowski, Ye Zhang, Zoltan Perko, Christopher Kurz, George Dedes, Guillaume Landry, Adrian Thummerer

**Affiliations:** ^1^ Department of Radiation Oncology LMU University Hospital, LMU Munich Munich Germany; ^2^ Division of Medical Physics in Radiation Oncology German Cancer Research Center (DKFZ) Heidelberg Germany; ^3^ National Center for Radiation Research in Oncology (NCRO) Heidelberg Institute for Radiation Oncology (HIRO) Heidelberg Germany; ^4^ Biophysics Department GSI Helmholtzzentrum für Schwerionenforschung Darmstadt Germany; ^5^ Department of Physics and Astronomy Heidelberg University Heidelberg Germany; ^6^ Institute of Biomedical Engineering Karlsruhe Institute of Technology Baden‐Württemberg Germany; ^7^ Department of Radiotherapy University Medical Center Utrecht Utrecht Netherlands; ^8^ Department of Radiation Oncology, Amsterdam UMC location Vrije Universiteit Amsterdam Cancer Center Amsterdam, Cancer Treatment and Quality of Life Amsterdam Netherlands; ^9^ Center for Proton Therapy, Paul Scherrer Institute (PSI) Villigen Switzerland; ^10^ Department of Radiation Oncology University Hospital Heidelberg Heidelberg Germany; ^11^ Radiation Physics, Department of Hematology, Oncology, and Radiation Physics Skåne University Hospital Lund Sweden; ^12^ Department of Radiation Science and Technology Delft University of Technology Delft the Netherlands; ^13^ Radformation Inc. New York USA; ^14^ Department of Medical Physics, Faculty of Physics Ludwig‐Maximilians‐Universität München Munich Germany; ^15^ German Cancer Consortium (DKTK), partner site Munich, a partnership between DKFZ and LMU University Hospital Munich Germany; ^16^ Bavarian Cancer Research Center (BZKF) Munich Germany; ^17^ Department of Radiation Oncology, Inselspital Bern University Hospital and University of Bern Bern Switzerland; ^18^ Department of Digital Medicine, Faculty of Medicine University of Bern Bern Switzerland

**Keywords:** dose calculation, dose prediction, deep learning, mri‐only

## Abstract

**Purpose:**

Accurate dose calculation is a fundamental component of modern radiotherapy, enabling precise tumor irradiation while sparing surrounding healthy tissue. With the increasing adoption of magnetic resonance imaging (MRI)‐guided radiotherapy and growing interest in real‐time adaptive radiotherapy, there is a demand for fast and accurate dose calculation techniques on both computed tomography (CT) and MRI. To facilitate research in this area, the DoseRAD2026 dataset and accompanying challenge introduce a publicly available benchmark dataset comprising paired CT and MRI data with corresponding beam‐level photon and proton Monte Carlo (MC) dose distributions, enabling the development and systematic evaluation of advanced dose calculation and prediction methods.

**Acquisition and validation methods:**

The DoseRAD2026 dataset comprises paired CT and MRI data from 115 patients (75 training, 40 testing) treated on a hybrid MRI‐linear accelerator (MRI‐linac) for thoracic or abdominal lesions and is derived from the SynthRAD2025 dataset. Image pre‐processing included deformable image registration, air‐cavity correction and resampling. Ground‐truth photon (6 MV) and proton dose distributions were generated using open‐source MC dose calculation algorithms and resulted in a training dataset of 40,500 photon beams and 81,000 proton beamlets.

**Data format and usage notes:**

The dataset is organized into photon and proton subsets, each comprising paired CT‐MRI images, beam‐level dose distributions, and JSON files specifying patient‐specific beam configurations. Data are provided in compressed MetaImage (.mha) format with standardized naming linking dose files to beam parameters in the JSON files. The dataset is released under a CC BY‐NC 4.0 license, with the training set available from April 2026 and the test set withheld until March 2030.

**Potential applications:**

The DoseRAD2026 dataset enables benchmarking and development of fast dose calculation methods for radiotherapy applications. Potential use cases include rapid beam‐level dose estimation for photon and proton therapy, MRI‐based dose calculation in MRI‐only and MRI‐guided radiotherapy workflows, and support for real‐time adaptive radiotherapy strategies.

## 1 INTRODUCTION

Radiation therapy is indicated for approximately half of all cancer patients, spanning curative, adjuvant, and palliative contexts.^1^, ^2^ Photon therapy, such as volumetric modulated arc therapy (VMAT)^3^, enables highly conformal dose distributions around the target volume through continuous gantry rotation combined with dynamic multileaf collimator (MLC) shaping, while sparing surrounding organs at risks (OARs). Proton therapy, such as intensity modulated proton therapy (IMPT)^4^, offers a physically distinct advantage through the Bragg peak^5^, whereby the majority of energy is deposited at a finite tissue depth, affording superior sparing of OARs distal to the tumor. In both modalities, the accuracy of dose calculation is foundational to treatment plan quality.^6^ As workflows increasingly move toward online (immediately prior to radiation delivery)^7^, ^8^ and ultimately real‐time (continuously during radiation delivery) adaptive radiotherapy^9^, ^10^, the computational demand placed on dose engines has grown substantially.

MC simulation^11^ on high quality CT is broadly accepted as the reference standard for dose calculation, owing to its accurate physics modelling of particle transport. However, general‐purpose MC implementations suffer from long computation times, with plan‐level runtimes spanning hours to tens of hours on a single central processing unit (CPU).^12^ Graphics processing unit (GPU) acceleration has substantially narrowed this gap for dose calculations, with recent GPU‐based MC engines reporting plan‐level runtimes in the range of a few to tens of seconds.^13^, ^14^, ^15^ In parallel, deep learning (DL) methods have emerged as a complementary strategy, demonstrating sub‐second dose prediction and showing early promise at MC‐comparable accuracy in both proton^16^, ^17^, ^18^, ^19^ and photon^20^, ^21^, ^22^, ^23^ therapy. Nevertheless, achieving MC‐level accuracy and the computational speed required for online or real‐time plan adaptation—ideally in the sub‐second or millisecond time‐frame—remains an open challenge.

MRI offers superior soft‐tissue contrast and eliminates ionizing radiation exposure compared to CT,^24^ making it valuable for target and OAR delineation as well as motion management in online adaptive radiotherapy. The clinical introduction of hybrid magnetic resonance imaging‐linear accelerator (MRI‐linac) systems^25^, ^26^ has enabled high‐quality soft‐tissue imaging prior to and during photon treatment delivery. Meanwhile, prototype systems integrating proton delivery with in‐room MRI, as well as MRI‐only proton workflows^27^, ^28^, have also been proposed. However, since MRI does not provide electron density or material composition information, dose calculation in both modalities continues to rely on CT. Current clinical practice bridges this gap via synthetic CT (sCT) generation^29^, ^30^, but this intermediate step introduces potential sources of dosimetric error(difference between sCT and CT), computational latency, and overhead to the dose calculation pipeline, remaining a bottleneck for true real‐time plan adaptation. Direct DL‐based dose prediction from MRI offers a conceptually cleaner alternative, collapsing the sCT intermediate into a single end‐to‐end inference step. Early investigations for both photon^31^, ^32^ and proton^33^, ^34^ modalities have demonstrated dosimetric feasibility, yet progress has been constrained by the absence of large‐scale paired datasets providing beam‐level dose ground truth on both CT and MRI.

To address this gap, we introduce the DoseRAD2026 dataset, the first publicly available external radiotherapy dose dataset providing paired CTs and MRIs (adapted from the SynthRAD2025 dataset^35^) alongside beam‐level photon (6 MV) and proton MC dose distributions generated using open‐source MC simulation code. The DoseRAD2026 challenge^36^ will utilize this dataset across four tasks: Task 1 (CT to photon dose) targets photon segment (the radiation beam shaped by a given MLC aperture) dose calculation on CT for VMAT delivery in thoracic and abdominal cases, where lung and bowel heterogeneities present a challenging test. Task 2 (MRI to photon dose) extends this to direct photon dose prediction on MRI, leveraging the paired CT‐MRI dataset to map MC doses calculated on CT onto MRI. Task 3 (CT to proton dose) addresses proton pencil beam dose calculation on CT across a range of proton energies in thoracic and abdominal cases. Task 4 (MRI to proton dose) targets proton dose prediction directly on MRI, following the same paired data as in Task 2. By releasing beam‐level dose labels, DoseRAD2026 is designed to support fast dose calculation both based on CT and also directly on MRI.

## 2 ACQUISITION AND VALIDATION METHODS

### 2.1 Dataset overview

The DoseRAD2026 dataset comprises paired CTs and MRIs and corresponding photon and proton dose distributions from patients with thoracic and abdominal malignancies. The imaging data were derived from Center B of the SynthRAD2025 dataset,^35^ a single‐institution subset of the public SynthRAD2025 challenge dataset originally released for synthetic CT generation. This subset was selected for the DoseRAD2026 challenge due to its favorable imaging characteristics. In particular, MRI acquisitions at center B were performed on an MRI‐linac using a standardized protocol, ensuring consistent patient positioning between CT and MRI as well as an axial field of view covering the full patient anatomy. In contrast, MRIs from other centers exhibited greater variability in acquisition protocols and imaging characteristics, and were sometimes acquired on diagnostic scanners.

For DoseRAD2026, an additional external dataset acquired on a similar MRI‐linac is used for testing, but these data will not be released publicly due to data protection restrictions. This article mainly covers the data from SynthRAD2025 Center B.

### 2.2 Image dataset

MRI–CT data from Center B of the SynthRAD2025 dataset originally comprised 140 thoracic and 140 abdominal patients, split 91/14/35 for training, validation and test sets, respectively. For the DoseRAD2026 training set, only the currently public SynthRAD2025 training cases were considered, while for testing, only patients from the currently private SynthRAD2025 test set were used, thereby avoiding any impact on the testing phases of both SynthRAD2025 and DoseRAD2026. Because Tasks 2 and 4 focus on direct MRI‐based dose calculation and thus impose strict requirements on CT–MRI alignment, all cases were manually reviewed in three orthogonal planes (axial, coronal, and sagittal) to assess deformable image registration quality (see Section 2.2.1), and cases with obvious misregistration were excluded to ensure high spatial correspondence between modalities. After screening, a total of 115 patients were selected for DoseRAD2026: 53 thoracic (39 training, 14 testing) and 62 abdominal cases (36 training, 26 testing).

#### 2.2.1 Pre‐processing

All SynthRAD2025 data had previously undergone several pre‐processing steps, including rigid registration, defacing, resampling, and cropping. A detailed description of the SynthRAD2025 pre‐processing pipeline is provided by Thummerer et al.^35^ For DoseRAD2026, four additional pre‐processing steps were performed.

First, CTs were deformably registered to the corresponding MRIs using the ConvexAdam deformable image registration framework.^37^ The ConvexAdam hyperparameters were manually tuned and are listed in the Supplementary Materials A.

Second, for abdomen cases, air cavity correction was applied to CTs based on MRI air segmentations. Air cavities in the deformed CTs were first identified by thresholding voxels below −200 HU and then filled with the median HU value of all neighboring voxels of the air cavity. Afterwards, air cavities segmented on the MRIs were transferred to the deformably registered CTs and filled with a constant value of −824 HU.^38^ To avoid unrealistically sharp edges at the air‐tissue interface, the transition between air and soft tissue HUs was slightly blurred in a 1‐voxel band around the air cavity boundary. This mimicked the original appearance of air cavities on CTs. MRI air cavity segmentation was performed using an nnU‐Net model^39^ trained on a subset of manually annotated cases (20 patients), and all predicted air cavity segmentations were subsequently reviewed and corrected manually.

Third, to reduce file sizes and MC photon dose computation times, CTs and MRIs for the photon tasks (Tasks 1 and 2) were resampled to $2\times 2\times 2\;{\mathrm{mm}}^{3}$, while images for the proton tasks (Tasks 3 and 4) were not resampled and remained on the same $1\times 1\times 3\;{\mathrm{mm}}^{3}$ grid as used for SynthRAD2025.

Finally, patient body contours were segmented on both CTs and MRIs using TotalSegmentator.^40^ The intersection of the two masks was used to define a common foreground region, and voxels outside this region were set to −1024 HU in CT and 0 in MRI.

#### 2.2.2 Image acquisition parameters

All center B MRIs were acquired on a ViewRay MRIdian 0.35 T MRI‐linac using a balanced steady‐state free‐precession sequence (bSSFP, TrueFISP), while CTs were predominantly acquired on a Canon Medical Aquilion/LB CT scanner. Tables 1 and 2 present detailed imaging parameters of CTs and MRIs of the respective datasets. Per patient imaging parameters are provided as metadata to the dataset release.

**TABLE 1:** MRI acquisition parameters for thorax and abdomen datasets (train/test splits).

	Thorax		Abdomen	
	Train	Test	Train	Test
**Manufacturer**	ViewRay			
**Model**	MRIdian			
**Field strength (T)**	0.35			
**Sequence**	Balanced steady‐state free‐precession (bSSFP, TrueFISP)			
**Acquisition**	2D, breath‐hold, no contrast			
**TE (ms)**	1.27 (84%)	1.27 (64%)	1.43 (53%)	1.43 (65%)
	1.43 (13%)	1.43 (29%)	1.62 (19%)	1.62 (19%)
	1.62 (3%)	1.62 (7%)	1.27 (17%)	1.44 (15%)
			1.44 (11%)	
TR (ms)	3.00 (84%)	3.00 (64%)	3.33 (53%)	3.33 (65%)
	3.33 (13%)	3.33 (29%)	3.83 (19%)	3.83 (19%)
	3.83 (3%)	3.83 (7%)	3.00 (17%)	3.36 (15%)
			3.36 (11%)	
**Flip angle (** 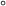 **)**	60	60	60	60
**Echo numbers**	1	1	1	1
**Number of averages**	1	1	1	1
**BW (Hz/px)**	604 (53%)	599 (43%)	537 (53%)	537 (65%)
	599 (32%)	537 (29%)	385 (19%)	385 (19%)
	537 (13%)	604 (21%)	599 (14%)	534 (15%)
	385 (3%)	385 (7%)	534 (11%)	
			604 (3%)	
**Acq. matrix**	310 × 360 (53%)	300 × 334 (43%)	266 × 266 (53%)	266 × 266 (65%)
	300 × 334 (32%)	266 × 266 (29%)	276 × 276 (19%)	276 × 276 (19%)
	266 × 266 (13%)	310 × 360 (21%)	300 × 334 (14%)	234 × 234 (15%)
	276 × 276 (3%)	276 × 276 (7%)	234 × 234 (11%)	
			310 × 360 (3%)	
**Phase enc. steps**	232 (53%)	225 (43%)	200 (53%)	200 (65%)
	225 (32%)	200 (29%)	207 (19%)	207 (19%)
	200 (13%)	232 (21%)	225 (14%)	176 (15%)
	207 (3%)	207 (7%)	176 (11%)	
			232 (3%)	
**Voxel spacing (mm)**	1.5 × 1.5 (97%)	1.5 × 1.5 (93%)	1.5 × 1.5 (81%)	1.5 × 1.5 (80%)
	1.6 × 1.6 (3%)	1.6 × 1.6 (7%)	1.6 × 1.6 (19%)	1.6 × 1.6 (19%)
**Slice thickness (mm)**	3	3	3	3
**Acq. time (s)**	25 (97%)	25 (93%)	25 (69%)	25 (65%)
	17 (3%)	17 (7%)	17 (19%)	17 (19%)
			23 (11%)	23 (15%)

*Note*: Voxel spacing is reported as in‐plane resolution; percentages indicate the proportion of scans with each value. For parameters with multiple values, values are listed in descending order of prevalence.

**TABLE 2:** CT acquisition parameters for thorax and abdomen datasets (train/test splits).

	Thorax		Abdomen	
	Train	Test	Train	Test
**Manufacturer**	Canon Medical	Canon Medical	Canon Medical	Canon Medical
	Aquilion/LB (97%)	Aquilion/LB	Aquilion/LB	Aquilion/LB
	Siemens			
	SOMATOM Drive (3%)			
**kVp**	120	120	120	120
**Exposure time (ms)**	500	500	500 (94%)	500
			750 (6%)	
**Tube current (mA)**	40 – 305	40 – 140	40 – 440	40 – 190
**Rows**	512	512	512	512
**Columns**	512	512	512	512
**Slice thickness (mm)**	3	3	3 (94%)	3
			5 (6%)	
**Voxel spacing (mm)**	1.4 × 1.4 (68%)	1.4 × 1.4 (50%)	1.4 × 1.4 (78%)	1.4 × 1.4 (92%)
	1.1 × 1.1 (29%)	1.1 × 1.1 (50%)	1.1 × 1.1 (17%)	1.1 × 1.1 (8%)
	1.0 × 1.0 (3%)		0.8 × 0.8 (3%)	
			0.6 × 0.6 (3%)	
**Data coll. diameter (mm)**	700 (68%)	700 (50%)	700 (78%)	700 (92%)
	550 (29%)	550 (50%)	550 (17%)	550 (8%)
	500 (3%)		500 (3%)	
			320 (3%)	
**Recon. diameter (mm)**	700 (68%)	700 (50%)	700 (78%)	700 (92%)
	550 (29%)	550 (50%)	550 (17%)	550 (8%)
	500 (3%)		418 (3%)	
			320 (3%)	
**Nr. of slices**	103 – 299	117 – 154	20 – 403	87 – 307

*Note*: Voxel spacing is reported as in‐plane resolution (isotropic); percentages indicate proportion of scans with each value.

#### 2.2.3 External test dataset

Additional test data were collected at an external institution, but are intended solely for the testing phase of the DoseRAD2026 challenge and will not be made publicly available. This dataset originally comprised 21 paired thoracic CTs and MRIs. Similar to the public data, MRIs were acquired on a ViewRay MRIdian 0.35T MR‐Linac using the same bSSFP sequence, a slice thickness of 3 mm and a pixel spacing of 1.6mm×1.6mm. CTs were acquired on a GE Medical Systems Discovery CT590 RT scanner, with a tube voltage of 120 kV, a tube current between 167 and 388 mA, an exposure between 12 and 49 mAs, a pixel spacing between 0.98 and 1.37 mm and a slice thickness of 2.5 mm.

These 21 cases underwent the same pre‐processing as described above and were manually screened for deformable image registration quality, yielding a filtered external test set of 7 thoracic patients. These data were acquired at a different institution, from a different patient cohort and on a different CT scanner, with MRI acquired on a separate ViewRay MRIdian 0.35 T MRI‐linac unit using the same bSSFP sequence. They therefore provide a controlled external test for DoseRAD2026 tasks.

#### 2.2.4 Image dataset split

Table 3 summarizes the dataset size and split for ease of reference.

**TABLE 3:** Patient distribution in DoseRAD2026 by anatomical region and dataset split.

	Publicly released			Not released
Region	Training	Testing	Total	External testing
Thoracic	39	14	53	7
Abdominal	36	26	62	—
**Total**	**75**	**40**	**115**	**7**

*Note*: Training and Testing are from SynthRAD2025 Center B and together constitute the publicly released dataset. The external testing cohort is used for the DoseRAD2026 challenge evaluation only and is not publicly released.

### 2.3 Dose dataset

After the pre‐processing and manual review, the corrected CTs were subsequently converted into mass density (details see Supplementary Materials B) and elemental composition maps using a scanner‐specific calibration curve.^41^ All MC simulations were implemented using the Geant4 toolkit (version 11.00‐patch‐03) with the QGSP_BIC_EMV physics list, scoring dose‐to‐medium of each voxel in units of Gray (Gy). Task‐specific simulation details for photon and proton beams are described in Sections 2.3.1 and 2.3.2.

#### 2.3.1 Photon MC simulation

VMAT plans were generated for all 75 training and 40 test patients using a VMAT implementation^42^ in the open‐source research treatment planning toolkit matRad^43^, ^44^ (v3.2.0). Each plan was optimized based on the patient's CT and clinically contoured structure set, using a simplified model configured for an Elekta Versa HD Linac with an Agility 160‐leaf MLC, in which jaw positions, leaf transmission, and tongue‐and‐groove effects were not modelled. Optimization followed a three‐stage sequential pipeline: fluence map optimization, leaf sequencing via the Siochi algorithm^45^ and direct aperture optimization.

The resulting plan comprised a single coplanar full arc (gantry $-180^{\circ }$ to $180^{\circ }$, couch 0°), yielding 181 control points from matRad, of which 180 were retained after removing the duplicate at $180^{\circ }$ (coincident with $-180^{\circ }$). Each control point was defined by a gantry angle, monitor unit (MU), and MLC leaf positions. 180 beam segments were then derived based on the MLC aperture of the corresponding control point. Each aperture was discretized into a binary mask on a uniform pixel grid (1mm×1mm), where a pixel was assigned a value of one if fully open and zero if blocked. For the 75 training patients, this pool of segments was augmented by randomly shifting each MLC leaf by ± 5 mm along its direction of travel two times, expanding the pool from 180 to 540 segments per patient (40,500 in total). VMAT plan segments from the 40 internal testing cases and 7 external testing cases were extracted without augmentation.

After segment extraction, Geant4 dose simulations with a 6 MV photon energy spectrum derived from the ELEKTA_PRECISE_6MV phase space files provided by the International Atomic Energy Agency^46^ were implemented (photon energy distribution in Supplementary Materials B). The virtual source point‐to‐isocenter distance was set to 100 cm. At the isocenter plane normal to the beam, a fully open segment mask would correspond to a 40cm×40cm field, and the segment mask was defined on a corresponding virtual plane of this dimension located at the opposite of the virtual source point. This served as a fluence source from which particles were emitted with focus on the virtual source point. The dose grid matched the patient CT spacing (2mm×2mm×2mm). A uniform 0.35 T magnetic field was simulated in Geant4, aligned parallel to the superior‐inferior patient direction and confined within a cylindrical region with a 50 cm diameter.^26^

For each training patient, the isocenter from the matRad VMAT plan was shifted along the superior‐inferior axis by −2, 0, and +2 cm, defining three arcs. For each gantry angle, the three variants in the 540‐segment pool (1 original + 2 MLC‐perturbed) were assigned to the three arcs, so that each arc received one unique aperture per angle (180 segments per arc). Segment doses were then simulated at gantry angles every $2^{\circ }$ for all three arcs, yielding 75patients×3arcs×180angles=40,500 training samples in total. Representative photon segment dose distributions in the training dataset for both Task 1 (CT) and Task 2 (MRI) are illustrated in Figure 1.

**FIGURE 1:**
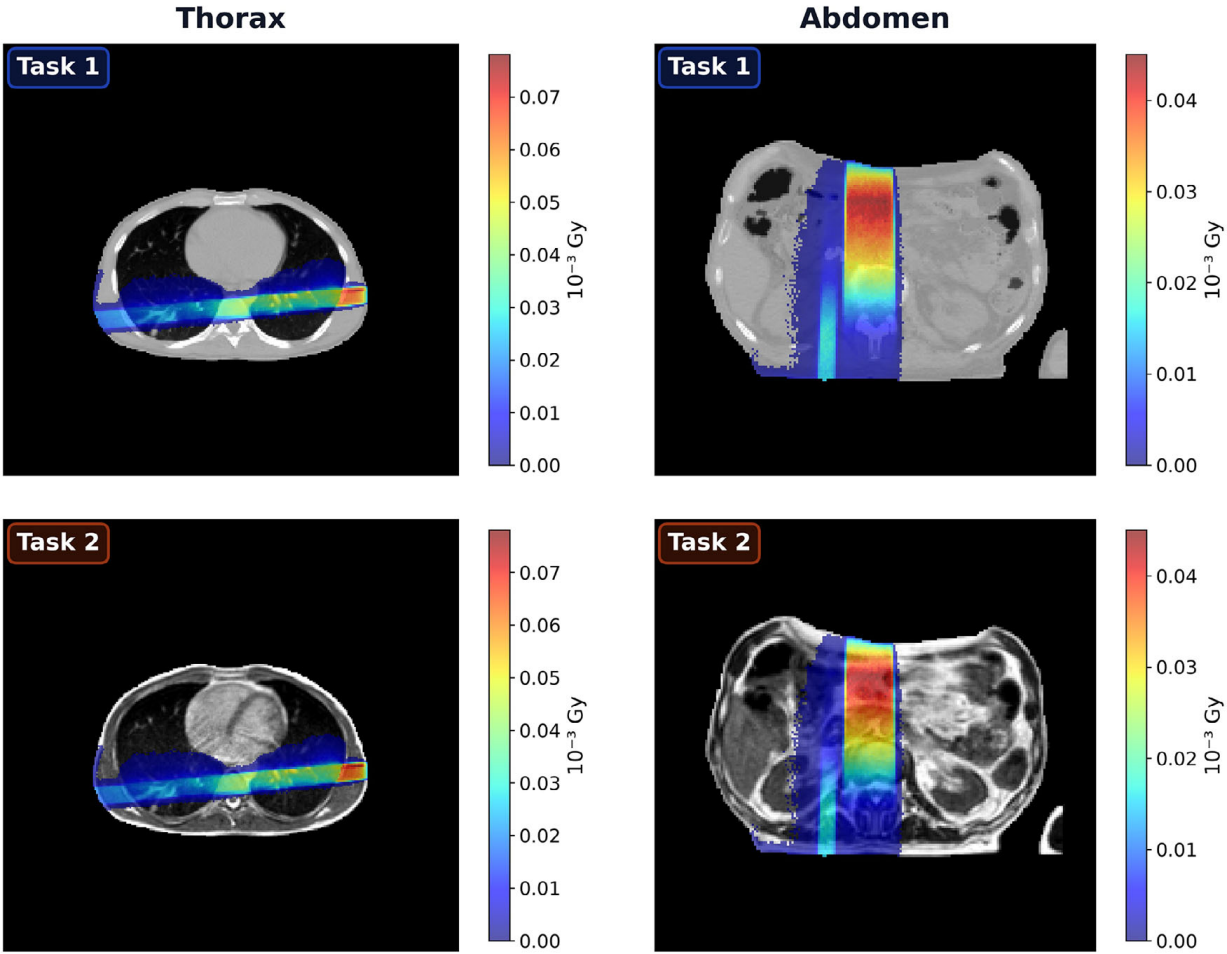
Representative photon segment dose distributions overlaid on CT (Task 1, top row) and MRI (Task 2, bottom row) images for thoracic (left column) and abdominal (right column) cases.

For the testing patients, segment doses were simulated according to each testing plan's control point sequence. Plan doses were then reconstructed by MU‐weighted accumulation of segment doses. The dose prescriptions to the planning target volume (PTV) were 70 Gy in 35 fractions for lung patients and 60 Gy in 30 fractions for abdomen patients (2 Gy per fraction in both cases). All MC testing plan doses were scaled so that $D_{95\%}\left(\text{PTV}\right)=0.95\times D_{\text{prescribed}}$. For both training and test sets, each photon segment dose was simulated at a fixed incident fluence of $1\times 10^{7}$ photons/${\mathrm{cm}}^{2}$, such that the number of simulated photons varies with segment size.

#### 2.3.2 Proton MC simulation

After the HU‐to‐mass density and elemental composition conversion, stopping powers were computed internally by Geant4 from the assigned material properties using the Bethe–Bloch formalism, with relative stopping power (RSP) (relative to water) optionally extracted in our Geant4 software (which may be useful for DL model training^47^). The initial proton energies ranged from 31.73 to 200.80 MeV (85 energy levels). A simplified proton beam model was employed to define the energy‐dependent beamlet energy spread (sigma from 0.78 to 6.11 MeV) and spot size (sigma from 4.00 to 7.99 mm), under a single‐Gaussian lateral approximation. Proton parameters were derived from matRad's native “Generic” proton machine and are listed in Table 4. The proton dose grid matched the patient CT spacing (1mm×1mm×3 mm) and no magnetic field was simulated.

**TABLE 4:** Proton beam model parameters: energy, energy spread ($\sigma_{E}$), and spot size ($\sigma_{s}$) for 85 energy levels (values rounded to 2 decimal places).

E (MeV)	$\sigma_{E}$ (MeV)	$\sigma_{s}$ (mm)	E (MeV)	$\sigma_{E}$ (MeV)	$\sigma_{s}$ (mm)	E (MeV)	$\sigma_{E}$ (MeV)	$\sigma_{s}$ (mm)
31.73	6.11	7.49	114.54	1.47	5.85	162.93	0.99	4.49
36.80	5.19	7.62	116.53	1.44	5.78	164.45	0.98	4.46
41.38	4.56	7.70	118.49	1.42	5.70	165.96	0.97	4.44
45.60	4.09	7.77	120.43	1.39	5.63	167.46	0.96	4.41
49.54	3.74	7.82	122.34	1.37	5.56	168.95	0.95	4.39
53.25	3.45	7.86	124.23	1.34	5.50	170.43	0.94	4.36
56.77	3.21	7.90	126.10	1.32	5.43	171.90	0.93	4.34
60.13	3.01	7.93	127.95	1.30	5.37	173.36	0.92	4.32
63.35	2.84	7.95	129.78	1.28	5.31	174.81	0.91	4.30
66.45	2.70	7.98	131.59	1.26	5.25	176.25	0.91	4.28
69.44	2.57	7.99	133.37	1.24	5.20	177.68	0.90	4.26
72.33	2.46	7.99	135.15	1.22	5.15	179.10	0.89	4.24
75.14	2.35	7.71	136.90	1.21	5.10	180.52	0.88	4.22
77.87	2.26	7.56	138.64	1.19	5.05	181.92	0.87	4.20
80.53	2.18	7.42	140.35	1.17	5.00	183.32	0.87	4.18
83.13	2.10	7.29	142.06	1.16	4.95	184.71	0.86	4.17
85.66	2.04	7.16	143.75	1.14	4.91	186.09	0.85	4.15
88.13	1.97	7.03	145.42	1.13	4.87	187.46	0.84	4.14
90.56	1.91	6.91	147.08	1.11	4.82	188.83	0.84	4.12
92.93	1.86	6.80	148.72	1.10	4.79	190.19	0.83	4.11
95.26	1.81	6.69	150.35	1.08	4.75	191.54	0.82	4.09
97.55	1.76	6.58	151.97	1.07	4.71	192.88	0.82	4.08
99.79	1.72	6.48	153.57	1.06	4.68	194.22	0.81	4.06
102.00	1.68	6.38	155.16	1.05	4.64	195.55	0.80	4.05
104.17	1.64	6.28	156.74	1.03	4.61	196.87	0.80	4.04
106.30	1.60	6.19	158.31	1.02	4.58	198.19	0.79	4.02
108.41	1.57	6.10	159.86	1.01	4.55	199.49	0.79	4.01
110.48	1.53	6.02	161.40	1.00	4.52	200.80	0.78	4.00
112.52	1.50	5.93						

For the 75 training patients, a total of 36 gantry angles were simulated in $10^{\circ }$ increments from $0^{\circ }$ to $350^{\circ }$ surrounding the isocenter. Along each gantry direction, proton spots were initialized as parallel pencil beams on a source plane located 100 cm upstream of the isocenter, approximating the extended virtual source‐to‐axis distances arising from the scanning magnet positions in clinical proton systems. At each gantry angle, 15 rays were arranged on a 5×3 beam's eye view (BEV) grid, with 5 fixed positions of {−40,−20,0,+20,+40} mm along the superior‐inferior axis, and 3 positions along the lateral axis consisting of 0 mm and two randomly sampled offsets from {±5k∣k∈[1,6]} mm. Each ray was assigned 2 beamlets of distinct energies sampled from Table 4, yielding 15×2=30 beamlets per beam. Across all 75 training patients, this setup produced 75patients×36angles×30beamlets=81,000 beamlet dose distributions as the training dose dataset. Representative proton beamlet dose distributions in the training dataset for both Task 3 (CT) and Task 4 (MRI) are illustrated in Figure 2.

**FIGURE 2:**
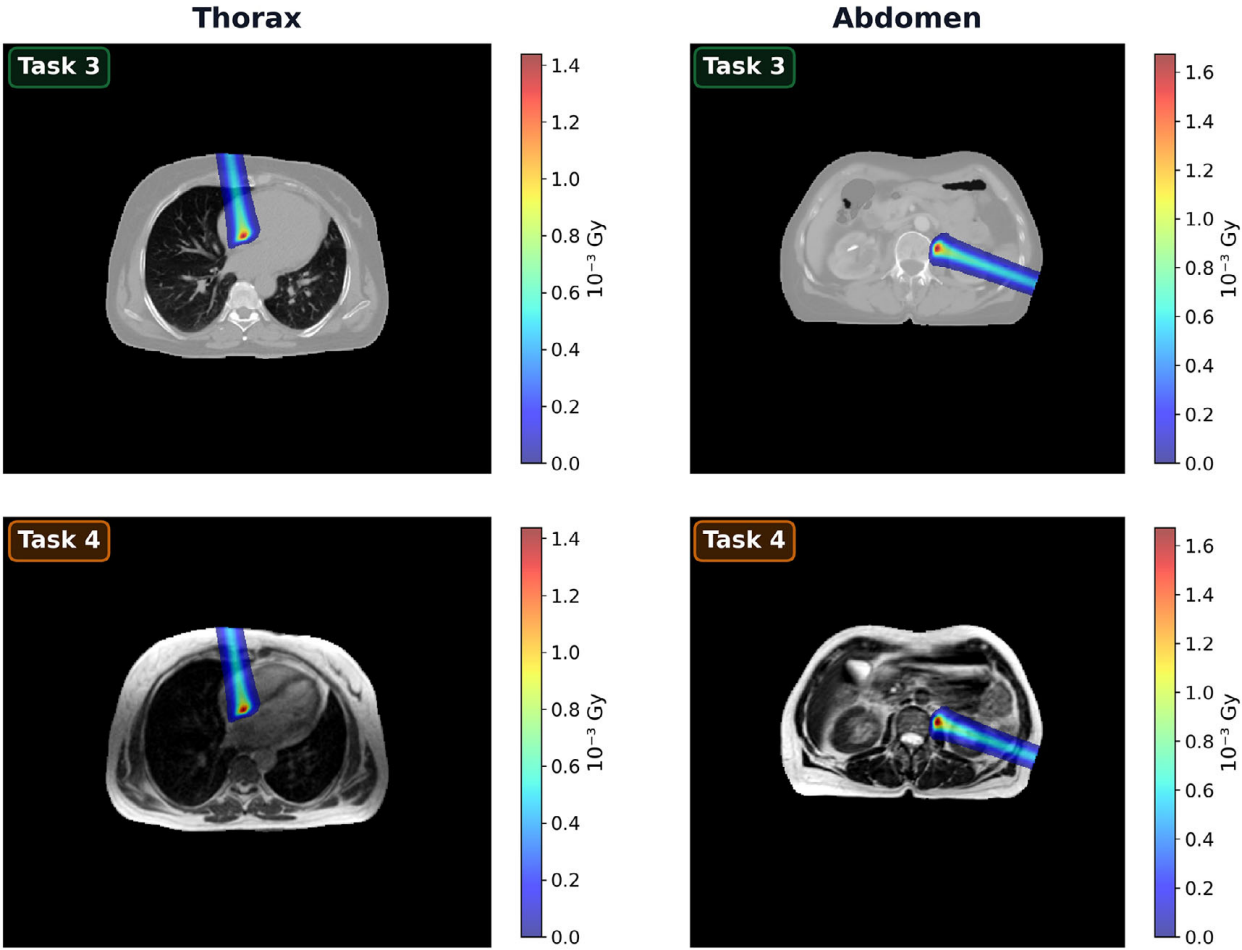
Representative proton beamlet dose distributions overlaid on CT (Task 3, top row) and MRI (Task 4, bottom row) images for thoracic (left column) and abdominal (right column) cases.

For the 40 testing patients, 40 IMPT plans were optimized based on the patient's CT and clinically contoured structure set using matRad. Plan doses were then calculated as a spot weight‐scaled superposition of beamlet doses using Geant4. The resulting plans comprise between 54 and 3101 beamlets each (mean 752). The PTV dose prescriptions were 70 Gy in 35 fractions for lung patients and 60 Gy in 30 fractions for abdomen patients (2 Gy per fraction in both cases). All MC testing plan doses were scaled so that $D_{95\%}\left(\text{PTV}\right)=0.95\times D_{\text{prescribed}}$. For both training and test proton dose simulations, each beamlet dose was simulated with $1\times 10^{6}$ primary protons.

#### 2.3.3 Post‐processing

All simulated photon and proton dose distributions were masked to zero outside the body contour derived from the deformed CT.

#### 2.3.4 Computational resources

The average Geant4 simulation time for a single photon segment dose was approximately 20–30 h on a single CPU core, while a single proton beamlet dose required approximately 2–3 h. All simulations were carried out on the computing clusters of the GSI Helmholtzzentrum für Schwerionenforschung, the Ludwig Maximilian University (LMU) Hospital Munich and Leibniz‐Rechenzentrum (LRZ) utilizing approximately 2000 CPU cores in total.

### 2.4 Data validation

#### 2.4.1 Image dataset validation

The DoseRAD2026 dataset was derived from the SynthRAD2025 dataset, which already underwent extensive quality control by the initial data providers through visual inspection to ensure completeness and overall data integrity. However, whereas the SynthRAD2025 dataset was designed to represent a heterogenous, multi‐center and multi‐anatomy cohort, containing a broad range of scanner models and acquisition protocols, the aims of the DoseRAD2026 challenge required special attention to image alignment between CT and MRI. This resulted in the inclusion of only a single center with data from two anatomical regions (thorax and abdomen). All included cases were manually screened and rigorously evaluated for high‐quality alignment between CTs and MRIs. This assessment was performed by two medical imaging experts, who visually inspected deformably registered image pairs with particular attention to alignment in bony structures, soft tissue interfaces, and external body contours. For these cases, detailed imaging parameters were systematically extracted from the SynthRAD2025 metadata and are provided as metadata to the DoseRAD2026 dataset to facilitate reproducibility and transparency.

The data inspection further revealed the presence of metal artifacts, including dental implants, in 17 out of 115 patients (13 in the training set and 4 in the test set). These cases were intentionally retained, as they provide a valuable opportunity to assess the robustness and sensitivity of different dose calculation approaches in the presence of clinically relevant imaging artifacts. A detailed list of the 13 patients with metal implants from the training set is provided in the Supplementary Materials C.

#### 2.4.2 Dose dataset validation

To assess MC statistical uncertainty, 10 independent simulations with different random seeds were performed on one representative thoracic and one representative abdominal case, for both photon segment doses and proton beamlet doses. The statistical uncertainty was quantified as the voxel‐wise standard deviation across the 10 repeated simulations, evaluated within dose regions exceeding 10% of $D_{\max}$ to exclude low‐dose voxels where relative uncertainty is inherently inflated. For photon segment doses, a primary fluence of $1\times 10^{7}$ photons/${\mathrm{cm}}^{2}$ yielded an average statistical uncertainty below 3% in both cases. For proton beamlet doses, simulating $1\times 10^{6}$ primary protons resulted in an average statistical uncertainty below 2%.

All photon dose distributions were verified using BEV extraction based on the beam direction to match the corresponding dose and visual inspection of the segment shape, whereas all proton dose distributions were checked via BEV extraction based on the beamlet direction.

### 2.5 Code availability

DoseRAD2026 preprocessing, MC simulation and matRad planning scripts are publicly available and listed in Table 5.

**TABLE 5:** Availability of DoseRAD2026 code and scripts.

Component	Repository URL
Pre‐processing scripts	https://github.com/DoseRAD2026/preprocessing
Geant4 simulation scripts	https://github.com/DoseRAD2026/geant4‐dose‐sim
Geant4 toolkit version	https://geant4.web.cern.ch/download/11.0.3.html
matRad VMAT planning scripts	https://github.com/DoseRAD2026/matrad‐vmat‐example
matRad commit used in this study	https://github.com/e0404/matRad/tree/71c95e96a1ecc7c7e025b9d75240ea009bdbc063
pyRadPlan pencil beam baseline	https://github.com/DoseRAD2026/pyradplan‐pb‐baseline

In addition, analytical pencil beam dose calculation scripts for both photon and proton modalities, built upon matRad's Python variant pyRadPlan^48^, are provided (link see Table 5). These scripts serve as templates for DoseRAD2026 challenge participants.

## 3 DATA FORMAT AND USAGE NOTES

### 3.1 Data structure and file formats

The DoseRAD2026 dataset is divided into two subsets, corresponding to photon and proton dose calculation tasks, respectively. Each subset comprises an image directory containing paired CTs and MRIs for each patient, a dose directory with dose maps for multiple beam configurations, and a plan JSON file specifying the associated beam parameters. The corresponding folder structures are illustrated in Figures 3 and 4. Each patient is identified by a unique alphanumeric identifier inherited from the SynthRAD2025 dataset.

**FIGURE 3:**
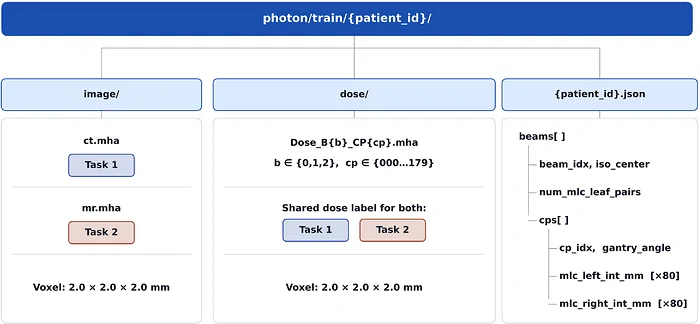
Folder structure for the photon dataset. Abbreviations: B, beam; CP, control point.

**FIGURE 4:**
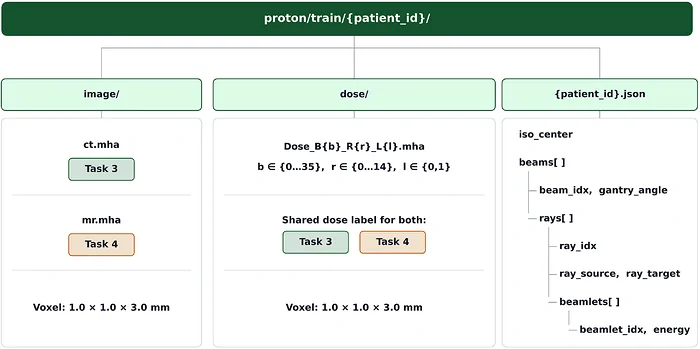
Folder structure for the proton dataset. Abbreviations: B, beam; R, ray; L, beamlet.

The dataset is distributed under a CC BY‐NC 4.0 International license. The training set was released in April 2026 and the test set will be released in March 2030, after the five‐year embargo period of the SynthRAD2025 test data expires. The data are hosted on Hugging Face, with a persistent DOI provided through Zenodo.^49^

### 3.2 Usage notes

Images and dose maps are provided as compressed MetaImage (.mha) files. These files can be accessed using the Insight Toolkit (ITK) framework or its simplified interfaces, such as SimpleITK, which offer bindings for multiple programming languages, including Python, R, Java, and C++. In addition, MetaImage files can be visualized using commonly available image viewers such as 3D Slicer, vv, and ITK‐SNAP.

The JSON file describes the corresponding beam configuration. For photon data, the JSON contains one or more beams (each associated with an isocenter position), with each beam comprising 180 control points defined by gantry angle and 80‐pair MLC leaf positions (mlc_left_int_mm, mlc_right_int_mm) relative to the beam central axis. For proton data, the JSON specifies the isocenter and 36 beam directions (gantry 0 to $350^{\circ }$, $10^{\circ }$ steps), with each beam containing rays defined by source and target coordinates, and the associated beamlet energies in MeV. Dose .mha files follow the naming convention Dose_B{beam}_CP{cp}.mha for photon and Dose_B{beam}_R{ray}_L{beamlet}.mha for proton, directly indexing into the JSON structure. A complete description of all JSON fields is provided in Supplementary Materials D.

The evaluation protocol for the DoseRAD2026 challenge, including metrics for dose accuracy and computational efficiency, and ranking scheme, is available in the MICCAI challenge description^36^ and on the challenge website (https://doserad2026.grand‐challenge.org/metrics‐and‐ranking/).

## 4 DISCUSSION

The DoseRAD2026 dataset is the first publicly available dataset providing paired CT and MRI alongside beam‐level MC dose ground truth for both photon and proton therapy, spanning thoracic and abdominal anatomies across four tasks. It aims to address two clinical needs. First, it provides a large‐scale benchmark dataset for DL‐based acceleration of MC dose calculation on CT, covering photon segment and proton pencil beam delivery in anatomical sites with challenging tissue heterogeneities. Second, by building on the SynthRAD2025 paired CT–MRI data^35^ with additional preprocessing steps including ConvexAdam deformable registration, air cavity correction and systematic manual quality control of CT–MRI alignment, DoseRAD2026 supports exploration of direct MRI to beam‐level dose calculation, offering a potential pathway toward real‐time MR‐guided or MR‐only adaptive radiotherapy. Together, the four tasks provide a common ground for benchmarking DL dose calculation methods across modalities, anatomies, and radiation types.

A key strength of the DoseRAD2026 dataset is the provision of beam‐level (rather than plan‐level) MC dose labels, which substantially increases the number of training samples per patient, enables plan dose accumulation, and allows flexible use of patient‐specific beam configurations by varying beam angles, isocenters, segment shapes, and proton energies independently. Dose labels for both photon and proton modalities are generated using Geant4, a well‐validated general‐purpose MC simulation framework, providing physically consistent ground truth and a broader scope than existing public datasets such as OpenKBP^50^, which covers head‐and‐neck photon plan doses only. The focus on thoracic and abdominal anatomies introduces clinically relevant tissue heterogeneities, including lung and abdominal air cavities, which present known challenges for analytical dose calculation methods. Full reproducibility is ensured through open‐source release of the preprocessing, Geant4 simulation, and matRad VMAT planning pipelines, together with an analytical pencil beam baseline, allowing the community to scrutinise and reproduce the data generation, or adapt them for their own research purposes.

A fundamental limitation of DoseRAD2026 is that both photon and proton dose distributions are generated using simplified, non‐clinical beam models. For photon plans, a generic machine model is used with binary MLC aperture masks, without modelling jaw positions, leaf transmission, or tongue‐and‐groove effects, and dose values are not calibrated to absolute clinical output. The VMAT plans are generated using matRad^43^, an open‐source research treatment planning toolkit, which ensures reproducibility but may not capture the full complexity of clinically optimized plans. For proton plans, an idealized generic beam model is employed rather than one specific to any clinical delivery system. Consequently, while the dataset is well‐suited for benchmarking DL dose calculation algorithms, the dose distributions are not directly representative of clinical deliveries. However, this trade‐off is accepted in favor of reproducibility and open‐source transparency. The Geant4 MC code and matRad planning toolkit ensures that data generation can be verified and extended by the community. Algorithms developed on DoseRAD2026 may nonetheless serve as a strong starting point for clinical translation: the generalized beam representations and physically consistent MC ground truth support the learning of fundamental dose–anatomy relationships, and fine‐tuning on machine‐specific data provides a practical pathway toward clinical deployment.

Another limitation is that all MRI data in the publicly released dataset originate from a single center and MRI‐linac system (0.35 T ViewRay MRidian), which may limit generalisability to other imaging platforms and patient populations. In addition, the dataset relies on deformable registration of CT to MRI to enable supervised learning for direct MRI‐based dose prediction. Despite careful validation and quality control, residual registration errors may persist. Since ground‐truth MC dose distributions were computed on CT images only and implicitly assumed to be spatially aligned with MRI, such misregistrations can impact MRI‐based tasks. Residual geometric MRI distortions following the vendor's corrections were not investigated in this dataset and masking the scans with a union of CT and MRI patient outline masks may further obfuscate their impact on dose calculations. These factors might limit the direct clinical applicability of models trained solely on the provided data and should be considered when interpreting the results or translating methods to clinical workflows. Furthermore, unlike the photon simulations, the proton simulations were performed without a magnetic field, corresponding to an offline‐MRI scenario. When the beam is delivered with the magnet on, Lorentz‐force beamlet deflection shifts the dose distribution and must be accounted for in dose calculation. As the released Geant4 pipeline supports magnetic‐field transport, we plan to include in‐field proton dose distributions at 0.35 T and higher field strengths in future dataset releases.

Beyond the DoseRAD2026 challenge, the dataset will remain publicly available to support continued development and benchmarking of fast CT‐ and MR‐based dose calculation methods. Future extensions may incorporate additional anatomical regions, multi‐institutional data, and more clinically realistic beam models to facilitate systematic evaluation of model generalizability and clinical applicability.

## 5 CONCLUSION

DoseRAD2026 introduces the first publicly available benchmark dataset, providing paired CT and MRI together with beam‐level photon and proton MC dose distributions for thoracic and abdominal radiotherapy. The dataset and accompanying challenge establish a common framework for the development and rigorous evaluation of fast dose calculation and dose prediction methods across imaging modalities, anatomies, and treatment modalities. We anticipate that the DoseRAD2026 dataset will serve as a valuable open resource for the community and help accelerate progress toward fast, accurate and robust (DL‐based) dose engines for CT‐ and MRI‐based dose calculation in adaptive radiotherapy.

## CONFLICT OF INTEREST STATEMENT

The Department of Radiation Oncology of the University Hospital of LMU Munich has research agreements with Brainlab, Elekta and ViewRay. Zoltan Perko is a Senior Applied Scientist at Radformation Inc.

## Data Availability

The DoseRAD2026 dataset is publicly available on Zenodo at https://zenodo.org/records/19347848.
